# Five year mortality and direct costs of care for people with diabetic foot complications are comparable to cancer

**DOI:** 10.1186/s13047-020-00383-2

**Published:** 2020-03-24

**Authors:** David G. Armstrong, Mark A. Swerdlow, Alexandria A. Armstrong, Michael S. Conte, William V. Padula, Sicco A. Bus

**Affiliations:** Southwestern Academic Limb Salvage Alliance (SALSA), Department of Surgery, Keck School of Medicine of University of Southern California, Los Angeles, USA

## Abstract

**Background:**

In 2007, we reported a summary of data comparing diabetic foot complications to cancer. The purpose of this brief report was to refresh this with the best available data as they currently exist. Since that time, more reports have emerged both on cancer mortality and mortality associated with diabetic foot ulcer (DFU), Charcot arthropathy, and diabetes-associated lower extremity amputation.

**Methods:**

We collected data reporting 5-year mortality from studies published following 2007 and calculated a pooled mean. We evaluated data from DFU, Charcot arthropathy and lower extremity amputation. We dichotomized high and low amputation as proximal and distal to the ankle, respectively. This was compared with cancer mortality as reported by the American Cancer Society and the National Cancer Institute.

**Results:**

Five year mortality for Charcot, DFU, minor and major amputations were 29.0, 30.5, 46.2 and 56.6%, respectively. This is compared to 9.0% for breast cancer and 80.0% for lung cancer. 5 year pooled mortality for all reported cancer was 31.0%.

Direct costs of care for diabetes in general was $237 billion in 2017. This is compared to $80 billion for cancer in 2015. As up to one-third of the direct costs of care for diabetes may be attributed to the lower extremity, these are also readily comparable.

**Conclusion:**

Diabetic lower extremity complications remain enormously burdensome. Most notably, DFU and LEA appear to be more than just a marker of poor health. They are independent risk factors associated with premature death. While advances continue to improve outcomes of care for people with DFU and amputation, efforts should be directed at primary prevention as well as those for patients in diabetic foot ulcer remission to maximize ulcer-free, hospital-free and activity-rich days.

Up to one-third of the half billion people with diabetes worldwide will develop a diabetic foot ulcer (DFU) over the course of their lifetime. Over half of DFUs will develop an infection. Of these, 17% will require an amputation [1–4]. Remarkably, people with diabetes fear amputation worse than death [5]. For patients who do not receive amputation and are able to heal their ulcer, 40% will develop a recurrence within 1 year, 65% within 5 years, and greater than 90% within 10 years [1, 6]. The greatest risk factor for a DFU is a previously healed DFU. These silent, sinister complications are now a leading cause of disability worldwide [7, 8]. Despite this high prevalence and morbidity, federal funding for studies related to DFUs remains at a 600-plus-fold disadvantage compared to other diabetes research in terms of public health impact. The disparity is even greater when compared to cancer research [9].

In 2007, we reported a summary of data comparing diabetic foot complications to cancer [10]. We thought that it might be appropriate to refresh this with the best available data as they currently exist. Since that time, more reports have emerged both on cancer mortality [11] and mortality associated with DFU, [12–14] Charcot arthropathy, [15–17] and diabetes-associated lower extremity amputation [18–27]. We collected data containing 5-year mortality from studies published after the previous publication in 2007 and calculated a pooled mean.

The mortality rate for people who undergo lower extremity amputation due to a DFU remains alarming: more than half of people with a major amputation will be dead in 5 years [21–25]. (Fig. 1). 5 year mortality for Charcot, DFU, minor and major amputations were 29.0, 30.5, 46.2 and 56.6%, respectively. This is even higher in people with concomitant chronic kidney disease and other comorbidities [25].

**Fig. 1:**
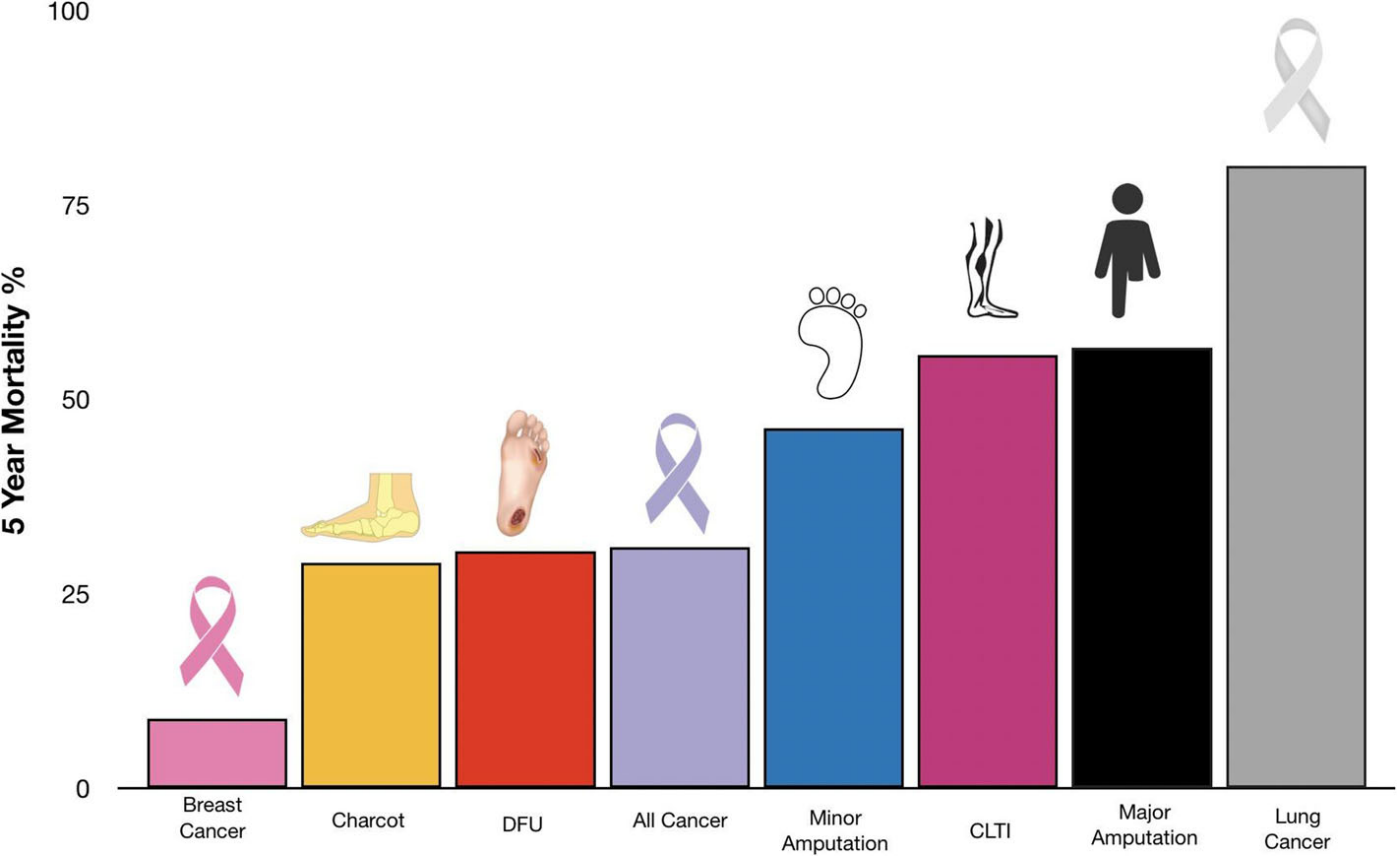
Five Year Mortality of Diabetic Foot Complications and Cancer. Diabetic foot complications compared to cancer. DFU = diabetic foot ulcers [11] = 30.5%. Charcot = Charcot neuroarthropathy of the foot [14]. All Cancer = pooled 5 year survival of all cancers [11]. CLTI = chronic limb threatening ischemia [28, 29]. Major Amputation = above foot amputation [20–22, 26, 27]. Minor Amputation = foot level amputation [17, 27]

Certainly, an important component of mortality in people with lower extremity complications of diabetes can be attributed to the severity of comorbidities with these patients often present - namely cardiovascular and renal disease worsened by reduced mobility [7]. This most certainly further reduces the attribution of cause away from lower extremity morbidity and toward a more familiar cardiovascular etiology. Indeed, people with a history of DFU have a life expectancy fully 5 years lower than age and disease-matched controls. The primary cause of death in these patients was listed as ischemic heart disease [30]. It is important to note, however, that, DFU and LEA appear to be more than just a marker of poor health. They are ***independent risk factors associated with premature death*** [31].

It is for these reasons that we have argued for a change in the syntax surrounding DFUs and other associated complications. Considering patients with healed DFUs as patients “in remission” rather than formally “healed” makes it easier for the patient, other clinicians, and policymakers to understand the possibility, or as the data suggest, probability, of a recurrence and to better communicate overall risk [1, 32]. It also indicates the need for regular follow-up and helps to prepare the patient for a lifetime of preventative management and mobility training [32]. With this mindset, patients can be properly educated about the dangers of diabetic foot disease and work towards maximizing ulcer-free, hospital-free, and activity-rich days, the same way a cancer survivor works to maximize cancer-free days [33, 34].

## The economic cost of DFUs

DFUs place a great economic burden on society, both to our healthcare system and due to lost productivity. In 2017, diabetes directly cost $237 billion in the USA, a 26% increase from 2012. On the order of one-third of these direct costs were attributable to care for diabetic foot disease [1, 35, 36]. In remarkable contrast, the 2015 direct costs for cancer in the USA were $80.2 billion - nearly equal to the attributable cost of diabetic foot disease [37]. As the number of people with diabetes is expected to rise over the coming decades we do not expect this cost or the rate at which it spirals out of control to slow down unless more serious measures are spent on preventive education and care. The U.S. National Cancer Institute’s budget is currently $6.4 billion to explore technological innovations in treatments and cures for cancer, and there should be a proportional response for diabetic foot disease [38]. Although patient education can play an important first step in the management of diabetic foot disease, new technologies are emerging which may help to reduce healing times, ulcer severity at clinical presentation, and overall costs.

Additionally, the emergence of remote patient monitoring technologies can allow us to predict and detect ulcers as or even before they form. Patients using a smart insole system, pressure-monitoring insoles transmitting real-time feedback to a smartwatch to cue offloading of sustained plantar pressure, in addition to the regular standard-of-care have been shown to have substantially lower rates of ulcer occurrence and approximately $15,000 less cost of care when ulcers do form, over a period of 18 months [39, 40]. In another study, a smart temperature-monitoring bathmat was able to detect DFUs 5 weeks before clinical presentation [41]. Further, in-clinic hyperspectral imaging devices have shown great promise in detecting wound formation weeks in advance and providing clinicians with useful information into the blood flow to current wounds, aiding in treatment decision-making [42]. Since the difference in cost between an early-stage DFU and a more severe DFU is at least an order of magnitude lower in almost every economy measured, [43] technologies that can help identify DFUs early or before they even form will be useful tools for patients, clinicians and health systems [44]. As these devices progress to be used in the home, along with other easy-to-use devices, we are hopeful that these technologies will help to alleviate the incidence of and costs associated with DFUs.

Altogether, these breakthroughs in technology and best-practice adherence offer providers and patients with economically dominant strategies to manage diabetic foot complications. Such strives are critical since the anticipated $80 billion currently being spent on diabetic foot disease is not sustainable. For merely pennies on the dollar, investment in prevention of this concerning complication represents a more economical practice and is better for the patient. Furthermore, monetary savings gained from the transition between treatment and prevention can be used to perpetuate further investments to address mitigating root causes of diabetes complications so that future patients suffer less.

In summary, complications associated with diabetic foot disease remain common, complex and costly. The economic impact of diabetic foot disease is comparable to cancer in every single way, but supportive technologies to predict and prevent onset offers healthcare potential savings in the short-run. Most importantly, focusing on early-stage preventative therapies and long-term supportive therapies for people in diabetic foot remission may yield both a greater lifespan and healthspan for the people we serve.

## Data Availability

Please contact author for data requests.
